# High Electromechanical Deformation Based on Structural Beta-Phase Content and Electrostrictive Properties of Electrospun Poly(vinylidene fluoride- hexafluoropropylene) Nanofibers

**DOI:** 10.3390/polym11111817

**Published:** 2019-11-05

**Authors:** Nikruesong Tohluebaji, Chatchai Putson, Nantakan Muensit

**Affiliations:** 1 Department of Physics, Faculty of science, Prince of Songkla University, Songkhla 90110, Thailand; 2 Center of Excellence in Nanotechnology for Energy (CENE), Songkhla 90110, Thailand

**Keywords:** electrostrictive properties, actuators, structural β-phase, dielectric properties, P(VDF-HFP) nanofibers, electrospinning, thermal compression

## Abstract

The poly(vinylidene fluoride-hexafluoropropylene) (P(VDF-HFP)) polymer based on electrostrictive polymers is essential in smart materials applications such as actuators, transducers, microelectromechanical systems, storage memory devices, energy harvesting, and biomedical sensors. The key factors for increasing the capability of electrostrictive materials are stronger dielectric properties and an increased electroactive β-phase and crystallinity of the material. In this work, the dielectric properties and microstructural β-phase in the P(VDF-HFP) polymer were improved by electrospinning conditions and thermal compression. The P(VDF-HFP) fibers from the single-step electrospinning process had a self-induced orientation and electrical poling which increased both the electroactive β-crystal phase and the spontaneous dipolar orientation simultaneously. Moreover, the P(VDF-HFP) fibers from the combined electrospinning and thermal compression achieved significantly enhanced dielectric properties and microstructural β-phase. Thermal compression clearly induced interfacial polarization by the accumulation of interfacial surface charges among two β-phase regions in the P(VDF-HFP) fibers. The grain boundaries of nanofibers frequently have high interfacial polarization, as they can trap charges migrating in an applied field. This work showed that the combination of electrospinning and thermal compression for electrostrictive P(VDF-HFP) polymers can potentially offer improved electrostriction behavior based on the dielectric permittivity and interfacial surface charge distributions for application in actuator devices, textile sensors, and nanogenerators.

## 1. Introduction

Electroactive polymers (EAPs) are intelligent materials that convert electrical energy to mechanical energy and vice versa. Common applications of such material include actuators, sensors, energy scavenging, etc. [1]. Electroactive polymers can be classified into two groups which depend on the mechanism responsible for actuation. Electronic EAPs comprise the first group, and the change in range is due to the driven electric field (ferroelectric polymers, dielectric EAPs, electroviscoelastic elastomers, electrostrictive polymers, piezoelectric polymers, etc.) [2]. The second group is composed of ionic EAPs, where the change in shape is due to the mobility or diffusion of ions and their conjugated substances (ionic polymers gels (IPGs), ionic polymer metal composites (IPMCs), conducting polymers, etc.). Electrostrictive polymers are one of the most common electronic EAPs that demonstrate a quadratically based relationship between the strain and electric field. This phenomenon is called electrostriction and occurs in all dielectric materials. It shows a large deformation of electric materials when the electric field is increased. The challenges for electrostrictive performance can be mitigated with a large induced strain under a low electric field. Therefore, improvement of the electrostrictive coefficient is necessary to achieve a high electric field-induced strain. In various studies, it has been suggested that the electrostrictive abilities of a polymer depend on the dielectric permittivity which is a significant parameter. Since the dielectric permittivity directly influences the achievable electrical field-induced strains in actuator applications, an improved electrostrictive polymer needs a high dielectric constant for achieving vast electric field-induced strains. Our previous papers have proposed that the interfacial charge or space charge distribution, which is referred to as a Maxwell–Wagner-type polarization of heterogeneous materials systems, can enhance the electrical and dielectric properties. The electrostriction effect can be observed in the polyurethane (PU) [3] and the family of PVDFs including poly(vinylidene fluoride-trifluoroethylene; P(VDF-TrFE) [4], poly(vinylidene fluoride-trifluoroethylene-chlorofluoro-ethylene); (P(VDF-TrFE-CFE)) [5,6], and poly(vinylidene fluoride-hexafluoropropylene (P(VDF-HFP)) [7].

Poly(vinylidene fluoride-hexafluoropropylene) is a semi-crystalline polymer with the linear formula (–CH_2_CF_2_–)*_x_*(–CF_2_CF(CF_3_)–)*_y_* and is a flexible, complex electroactive hydrofluorocarbon polymer that has well-established dielectric properties. Moreover, the features of P(VDF-HFP) are non-toxicity, high stability, its shape and size tailoring ability, and recycling aptitude; these copolymers are gaining momentum in widespread actuator technologies [1]. According to prior literature, Xiaoyan Lu [2] reiterated the strain response in P(VDF HFP) film, and a content of 5% and 15% HFP was measured for electric fields of 0–55 MV/m. Poly(vinylidene fluoride-hexafluoropropylene) can crystallize into α, β, and γ phases [3]. The most common and dominant phase among the three phases, the β-phase, has an orthorhombic structure and an all trans (TTTT) molecule zig-zag conformation. In the β-phase, all the dipoles are aligned in the same direction. It has the most exceptional spontaneous polarization per unit cell which is related to its dielectric properties [4,5]. However, it is challenging to obtain β-P(VDF-HFP). There are more conventional techniques for improving polar β-P(VDF-HFP) which involves electrical poling [6], mechanical extension (drawing) [7], melting processes at high pressure [8], mixing and blending with groups of fillers such as ceramic, clay, or montmorillonite (MMT) [9], and groups of hydrated ionic, magnesium chloride hexahydrate (MgCl_2_·6H_2_O) [10], Ni(OH)_2_ nanoparticle [11] and groups of conductive nanoparticle, including multiwalled carbon nanotubes (MWNTs) [12,13], carbon nanotubes CNTs [14] and graphene [15]. For example, Swagata Roy et al. [11] presented that the large β-phase of P(VDF-HFP)/MMT up to 85.22% and 82.1% from P(VDF-HFP)/NiMMT composites films. This effect increased the dielectric constant of the P(VDF-HFP)/MMT, and the P(VDF-HFP)/NiMMT film increased due to the increase in MMT content in the matrix of the polymer which exhibited a large interfacial area per unit volume. This is associated with the interfaces of the clay particles and the polymer chains that develop [11]. This result explains that, along with the strong interaction formed between the positive –CH_2_ dipoles and the negatively charged surface of MMT affection, the vital heightening of the equate that localized the polarization was accompanied with the filler as well as the coupling between the adjoining grains. In addition, the different nanofillers, magnesium chloride hexahydrate (MgCl_2_·6H_2_O), and stretching nanocomposite films also had an effect. The results showed that the P(VDF-HFP)/MgCl_2_·6H_2_O nanocomposite films, which stretched four-fold, achieved 90% of the β-phase. This experience arises from hydrogen bonding between the ionic interactions and unit chain of the polymer with the hydrated Mg–salt and the polar solvent. Various studies have been undertaken to improve the polar β-phase with the incorporation of conductive elements, for example, MWNTs, CNTs, and graphene [15,16,17,18,19,20,21]. The report by Zhou et al. [22] demonstrated the β-phase of P(VDF)/graphene composite nanofibers increased with an increase of graphene 0.1 wt %. The side effects of graphene nanomaterials have improved the stretching effect in the phase transformation of P(VDF) nanofibers. The key factors enhancing the electrostrictive abilities of P(VDF-HFP) are its electroactive β-phase and dielectric permittivity. It was found that P(VDF-HFP) improves the dielectric permittivity because of the intense polarization under an electric field [23]. In this work, we demonstrated the electrostriction of P(VDF-HFP) nanofibers because of its excellent dielectric properties, high surface-area-to-volume ratio, and highly crystallinity. Moreover, the electrostrictive properties of P(VDF-HFP) nanofibers are innovative and worth focusing on. These electrical properties are directly related to permittivity and phase transformation which strongly depend on the surface charge distributions of the material. Increasing the dielectric constant and electroactive β-phase content enhances the electrostrictive coefficient [24]. If the electroactive polymers based on electrostrictive effects include a high dielectric constant, it will likely produce a strong polarization contribution when inducing the external electric field; this generates their large electromechanical deformation. Large electromechanical deformations based on electrostrictive behavior occurred in the high dielectric polymers under their induced polarization contributions when increasing the external electric field strength.

The obtained dipole–dipole interactions in a previous study gave rise to large electrostriction [25]. In this work, we used an electrospinning and thermal compression method to change the geometric morphology of the phase distribution and increase interfacial surface charge distributions.

The selection of the electrospinning condition, including fiber orientation, also supports varying degrees of crystallinity and phase content [26]. It can provide self-induced orientation and electrical poling which increase the electroactive β-crystal phase and dipolar orientation at the same time. In fact, electrospinning can essentially provide a high surface-area-to-volume ratio in electrospun membranes. Several studies have reported that the interfacial charge or space charge distribution, which is referred to as a Maxwell–Wagner–type polarization, can enhance the electrical and dielectric properties in heterogeneous systems [12]. It was found that grain boundaries and interfaces among two regions within a material frequently give rise to interfacial polarization. Thermal compression-induced interfacial polarization occurs owing to the accumulation of interfacial surface charges between two β-phase regions in P(VDF-HFP) fibers. 

Therefore, this work set out to study the effects of combining electrospinning and thermal compression of electroactive P(VDF-HFP) nanofibers on their microstructure, crystallinity, β-phase, thermal properties, mechanical properties, electrical and dielectric properties, and, also, their electrostrictive properties with a view to apply them in actuators, textile sensors, nanogenerators, and nanoelectronic devices.

## 2. Experimental

### 2.1. Materials 

**Figure 1:**
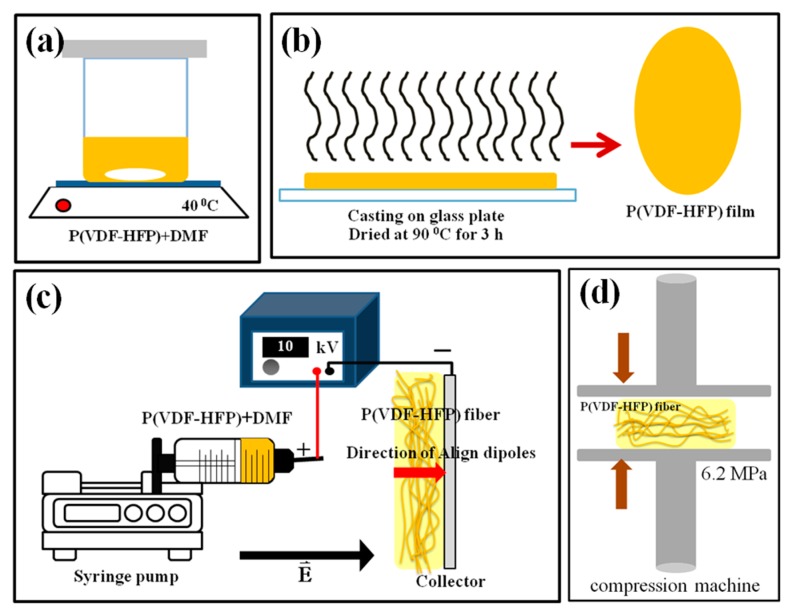
Schematic of the synthesis used with all samples. (**a**) Homogeneous poly(vinylidene fluoride-hexafluoropropylene) (P(VDF-HFP) solution, (**b**) solution casting, (**c**) electrospinning, and (**d**) fibrous film compression.

The P(VDF-HFP) powder with 10 wt % HFP (427179, Sigma–Aldrich, Washington, DC, USA) was used as the matrix. The solvent was *N,N*-dimethylformamide (DMF) (D158550, Sigma–Aldrich, Washington, DC, USA). The P(VDF-HFP) solution was prepared as follows. Firstly, 25 wt % of dried P(VDF-HFP) pellets were dissolved in the DMF solvent. The mixture was stirred at 40 °C using a magnetic stirrer in order to achieve a homogeneous solution, as shown in Figure 1a.

### 2.2. Synthesis of P(VDF-HFP) Films

The P(VDF-HFP) film was fabricated by solution casting (Figure 1b). The P(VDF-HFP) solution was poured onto a glass plate and dried at 80 °C for 3 h. In the end, the P(VDF-HFP) film was formed on the glass substrate.

### 2.3. Synthesis of P(VDF-HFP) Fiber

The P(VDF-HFP) fiber mats were fabricated by electrospinning (Figure 1c). The viscous P(VDF-HFP) solution was loaded into a 20 mL plastic syringe connected to a stainless-steel needle (gauge 20.5) and fed with a syringe pump (Nz1000 NEWERA Pump Systems Inc., New York, NY, USA) at a flow rate of 1.0 mL h^−1^. An electric field was generated by a high voltage supply (Trek model 610E, New York, NY, USA), capable of up to 10 kV DC. The positive pole was connected to the steel syringe needle and the collector aluminum plate was the grounded target 11 cm away from the tip of the needle. Porous fibrous films were obtained on the collector plate and were dried overnight at room temperature to evaporate the rest of the solvent. Finally, the fibrous film was compressed with a compression machine (Chareon tut, PR2D-W00L350-PM-WCL-HMI, Samutprakarn, Thailand) at 6.2 MPa and a selected temperature (30, 60 or 80 °C) for 10 min, as shown in Figure 1d. The thickness of all samples was measured using a thickness gauge, and it was in the range of 200 ± 50 μm. 

### 2.4. Material Characterization

#### 2.4.1. Surface Topography

The structure and morphology of the P(VDF-HFP) film and fibers were determined by scanning electron microscopy (SEM, FEI Quanta 400, Netherlands). All samples were sputter-coated with gold prior to the SEM imaging. The average fiber diameter and porosity of each sample was analyzed by ImageJ software (National Institutes of Health, 1.46, Madison, WI, USA).

#### 2.4.2. Crystalline Structure and Phase Investigation

The crystalline structure in all samples were examined using an X-ray diffractometer (XRD; X′Pert MPD, Philips, Netherlands) in the 2θ range from 5° to 90° at the scan rate 0.05° s^−1^ using Cu-K_α_ radiation (wavelength 0.154 nm) under a voltage of 40 kV. The crystallinity X_c_ can be estimated as follows [27]:

(1) $$X_{c}=\frac{\Sigma A_{cr}}{\Sigma A_{cr}+\Sigma A_{amr}}\times 100\%$$

where $\Sigma A_{amr}$ and $\Sigma A_{cr}$ are the total integral areas of amorphous halo and crystalline diffraction peaks, respectively. The α and β-phase contents were elucidated from IR spectra obtained with a Fourier transform infrared spectrometer (FTIR-8400S, Shimadzu, Tokyo, Japan). The absorbance data for all samples covered the wavenumber range 400–1000 cm^−1^ with a resolution of 4 cm^−1^. The fraction of β-phase, F(β) in films or fibers, was calculated using the Lambert–Beer law [10]:

(2) $$F\left(\beta \right)=\frac{A_{\beta }}{\left(\frac{K_{\beta }}{K_{\alpha }}\right)A_{\alpha }+A_{\beta }}=\frac{A_{\beta }}{1.26A_{\alpha }+A_{\beta }}$$

where A_α_ and A_β_ are the absorbance at 764 and 840 cm^−1^, respectively. K_α_ = 6.1 × 10^4^ cm^2^ mol^−1^ and K_β_ = 7.7 × 10^4^ cm^2^ mol^−1^ are the absorption coefficients at 764 and 840 cm^−1^, respectively.

The absolute β fraction (%β)is obtained from F(β) and X_c_, as in [28]:

(3) $$\%\beta =F\left(\beta \right)\times X_{c}$$

#### 2.4.3. Thermal Analysis

The thermal characteristics melting temperature (*T*_m_), crystallization temperature (*T*_c_), enthalpy of melting (ΔH_m_), and enthalpy of crystallization (ΔH_c_) were investigated using a differential scanning calorimeter (DSC, Perkin Elmer DSC7, USA). All samples were heated from −100 to 160 °C at a heating rate of 5 °C/min under the N_2_ atmosphere. Thus, a mass around 10 mg was sealed in an aluminum crucible for DSC-operating experiments.

#### 2.4.4. Mechanical Analysis

The mechanical material properties storage modulus (*E*′), loss modulus (*E*) and tan delta (tanδ) were characterized in tensile mode by dynamic mechanical analysis (DMA, Perkin Elmer, Waltham, MA, USA). The elastic behavior is observed from the storage modulus. Tan delta was the ratio of loss modulus to storage modulus. It is often called damping and informs about the energy dissipation in a material. The β-relaxation can be found at −50 °C which corresponds to the segmental motions in the amorphous phase. Also, the α-relaxation is detected above room temperature. The elastic properties of the material and for short-term creep can be recognized from this relaxation [29]. The DMA testing was performed on a temperature ranging from −110 to 160 °C at a heating rate of 5 °C/min with an oscillatory stress amplitude of 0.1 MPa and frequency of 1 Hz to provide the viscoelastic response.

#### 2.4.5. Electrical Properties

The electrical properties, specifically the dielectric constant (${\varepsilon^{\prime }}_{r}$) which is the real part of the relative permittivity, electrical conductivity (σ), and dielectric losses (tanδ) were evaluated with an LCR meter (IM 3533 HIOKI, Japan) in the frequency range 10^0^–10^5^ Hz. Each sample, with a thickness of approximately 200 ± 50 μm, was placed among two indium tin oxide electrodes (1 cm diameter) and supplied with 1 V. The dielectric constant can be calculated from [17]:

(4) $${\varepsilon^{\prime }}_{r}=\frac{Ct}{\varepsilon_{0}A}$$

where *C*, *t*, *A*, and ε_0_ are the capacitance of the sample, thickenss, the contact area of the electrode, and permittivity of free space (8.854 × 10^−12^ Fm^−1^), respectively. The electrical conductivity σ is calculated using the equation:

(5) $$\sigma =G\left(\frac{t}{A}\right)=2\pi f\varepsilon_{0}{\varepsilon^{\prime }}_{r}\tan\delta$$

where *G* is the conductance and *f* is the applied frequency. The dielectric loss tangent (tanδ) can be estimated from the relationship:

(6) $$tan\delta =\frac{{\varepsilon^{\prime \prime }}_{r}}{\varepsilon \prime_{r}}$$

Here, ${\varepsilon^{\prime \prime }}_{r}$ is the imaginary component of relative permittivity.

#### 2.4.6. Electrostrictive Properties

**Figure 2:**
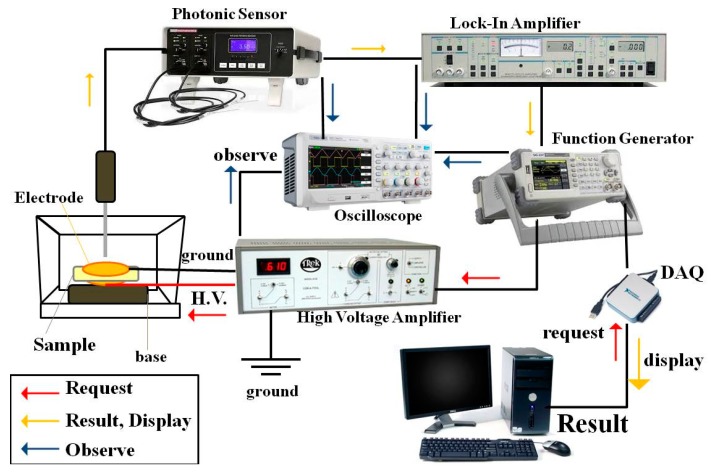
The electrostriction setup.

The electrostrictive property of the sample was evaluated by measuring the deformation of strain-induced at low frequency and low electric field strength (f = 1 Hz, *E* ≤ 3 MV/m) using the photonic displacement apparatus (MTI-2100 Fotonic sensor, New York, USA, sensitivity 5.8 μm/V) setup demonstrated in Figure 2. The dimension of the sample was 3 × 3 cm^2^ and the same thickness of 300 μm. The sample was sandwiched among brass electrodes (diameter 2 cm). The electric field (E_3_) of a high-voltage power supply (Trek model 610E, New York, USA) was applied along the thickness direction of the sample, which was the so-called “3” direction. The electric field-induced strain in polarizable materials was measured in the same direction and, hence, denoted as S_3_, then the electrostrictive coefficients (M_33_) were given, the relationship can be expressed according to the equation:

(7) $$S_{3}=M_{33}E_{3}^{2}$$

The electrostrictive coefficient can be calculated from the slope of the strain (S_3_) versus the square of the electric field (E^2^_3_). As a consequence, it can be expressed according to Equation (7).

## 3. Results and Discussion

### 3.1. Structure and Morphology 

**Figure 3:**
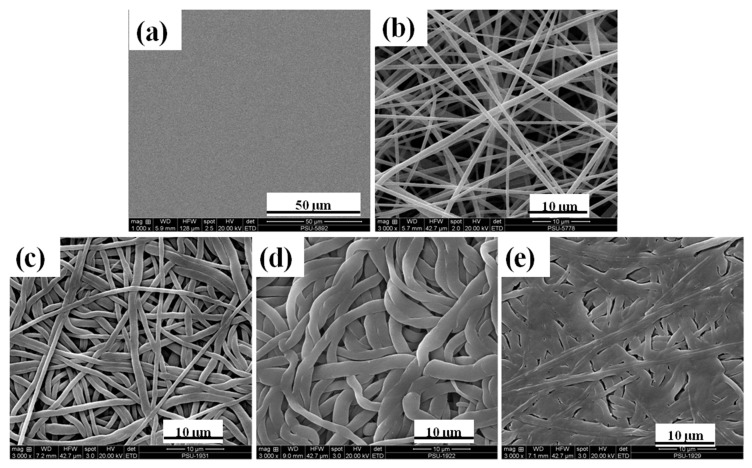
SEM images of the P(VDF-HFP) (**a**) film, (**b**) fiber, (**c**) 30 °C compressed fibers, (**d**) 60 °C compressed fibers, and (**e**) 80 °C compressed fibers.

In Figure 3, SEM micrographs display the morphology of P(VDF-HFP) film and fibers. Figure 3a presents the surface of the P(VDF-HFP) film with a 50 μm scale bar showing a clearly smooth, homogeneous, and non-porous surface. The electrospun membranes show a random orientation distribution, high porosity, and smooth and bead-free fibers with an average diameter of 600 ± 50 nm in Figure 3b. Figure 3c–e shows the electrospun P(VDF-HFP) nanofibers after compressing at 30, 60, and 80 °C, respectively. The compression at an elevated temperature reduced porosity and flattened fibers, with an apparent increase in diameter. In Figure 3e, the surface of the fiber mat was almost similar to the film, but with some porosity remaining. The fibers had large contact surfaces which is good for exchanging electric charges. Nanofibers have potential use in the production of sensing devices, because they have a large specific surface that enhances their sensitivity as a sensor [7]. In a previous paper, Kang et al. [30] studied the effect of load compression parameters with electrospun nanofibers at different types of polymers. They compressed poly(caprolactone); PCL and poly(vinyl alcohol); PVA and polyurethane; and PU and nylon nanofibers using a KES-G5s compression tester at different loads (0.5, 1, and 2 N, respectively) under room temperature. They explained that the movement of the fibers was related to the inter-fiber frictions when obtained fibers were passed. The changed density and loss of space between the layers occurred under an applied force. However, the morphology and structure of fibers under compression are not only magnitude force and direction force but also density materials, frictions of fibers, and operating temperature. In our case, the temperature conditions of 30, 60, and 80 °C for P(VDF-HFP) fiber mats were studied under a certain compression force. The morphology of P(VDF-HFP) film and fiber mats depends on the temperature effect, shown in Figure 3, and the changing temperature conditions related to the modification of the interfacial effect within P(VDF-HFP) film and fiber mats. Under the testing conditions, the P(VDF-HFP) fiber mats were strongly melted when the operating temperature increased due to the reduction of the glass temperature.

### 3.2. X-ray Diffraction (XRD) Analysis

**Figure 4:**
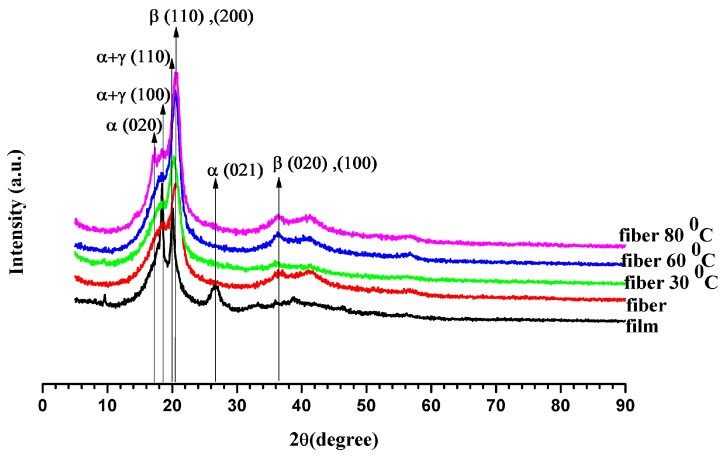
X-ray diffractograms for P(VDF-HFP) film, fiber, and fiber mats compressed at 30°, 60°, and 80 °C.

To confirm the presence of β-phase crystals in P(VDF-HFP) fibers, XRD analysis was performed. Figure 4 displays the XRD patterns generated for the P(VDF-HFP) film, fiber, and fiber mat compressed at 30, 60 or 80 °C. The characteristic reflections of the crystalline phases were seen in the XRD spectrum at 2θ = 17.9° (020) and 26.8° (021) which indicate the large spherulites of the non-polar α-phase crystals while 2θ = 18.5° (110) and 20.1° (110) correspond to the smaller spherulites of γ-phase crystals that co-exist with the α-phase. The specific peaks at 2θ = 20.3° (110) and (200) and 36.7° (020) and (100) correspond to β-phase diffraction [31]. The P(VDF-HFP) film shows the largest non-polar α peak at 26.8° and the smallest β peaks at 20.3° and 36.7°, because it lacks the electrical poling and mechanical stretching treatments. Thus, it shows the α-phase that is commonly the dominant phase in P(VDF-HFP) [32]. After electrospinning, the P(VDF-HFP) fibers and compassed fibers showed a strong β peak (110) at 2θ = 20.3° for the β-phase having an all-trans (TTTT) conformation, while the β peak (020) at 2θ = 36.7° was not clearly observed. Normally, the magnitude of this β peak (020) was quite small when compared with the β peak (110) which may be attributed to the formation of crystalline region and order of polymerization. Therefore, in our work, it was necessary to continue studying by Fourier transform infrared (FT-IR) analysis. It was apparent that the electrospinning successfully formed β-phase crystallites; this should enhance the electrostrictive properties of the nanofibers. The high voltage used during the electrospinning aligned the electric dipoles in the P(VDF-HFP) solution, and the degree of alignment was determined by the applied electric field [23]. In addition, the fibers after pressure and annealing treatments increased the crystalline and β-phases. This demonstrates that the formation of β-phase was induced by electrospinning, pressing, and annealing. 

Furthermore, the crystallinity, X_c_, of the film and fibers are presented in Table 1. The X_c_ increased from 49.47% to 55.03% with the compression temperature as shown by the strong peaks for β. The increased crystallinity could be due to the active interactions of the surfaces with polymer chains, inducing the formation of polar β-polymorphs from non-polar α spherulites. In other words, the fraction of crystalline material gradually increased. This appeared to be inferior to the α- to β-phase transformation, since the β-phase strongly depends on the overall crystallinity. However, using only XRD analysis is not enough for comparing the degrees of β-phase transformation, because the α and β peaks are close to each other and the changes are not clear. Therefore, Fourier transform infrared (FT-IR) analysis can provide additional data on the phase structure.

### 3.3. Fourier Transform Infrared Spectroscopy 

**Figure 5:**
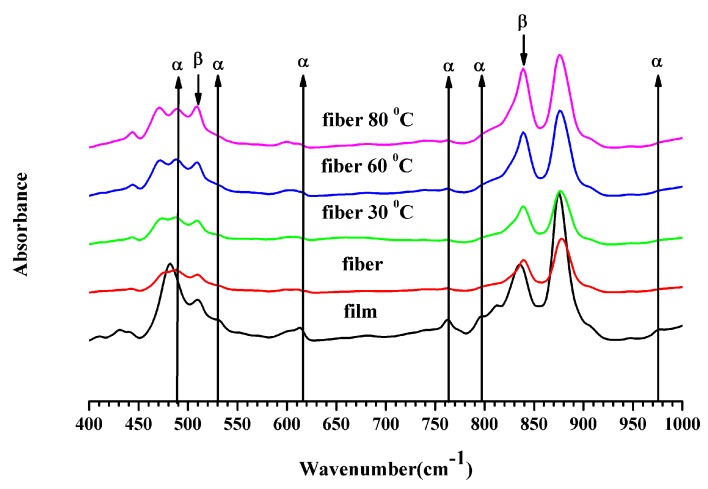
IR spectra of film and fiber P(VDF-HFP) for wavenumbers from 400 to 1000 cm^−1^.

The FTIR spectra are displayed in Figure 5. These were employed to assess the α- and β-phases, the F(β) of the crystalline region and %β in the samples. According to the literature [32], the non-polar α-phase in P(VDF-HFP) is detected in absorbance bands around 490 cm^−1^ (−CF_2_ wagging), 530 cm^−1^ (−CF_2_ bending), 615 cm^−1^ (skeletal bending), 764 cm^−1^ (−CF_2_ bending), 795 cm^−1^ (−CH_2_ rocking), and 975 cm^−1^ (twisting) in the IR spectra. In contrast, the large absorbance peaks of the β-phase, attributed to the electroactive polar β-polymorph with a parallel dipole moment, were found at cm^−1^ (−CF_2_ stretching) and cm^−1^ (−CH_2_ rocking, −CF_2_ stretching, and skeletal C−C stretching) in the spectrum.

In the FTIR spectrum, the P(VDF-HFP) film presented the most α-phase at 490 and 764 cm^−1^. If comparing the IR spectra of the film and nanofiber in Figure 5, it showed a shift of the IR peak of the P(VDF-HFP) film which had a lower wavenumber. The normal α-phase of the P(VDF-HFP) film presented the spectrum peak of −CF_2_ wagging or out of plane bending and positioned at 490 cm^−1^ [32]. These results are due to the stress and variation in the morphology [33]. Moreover, it may be attributed to a reduction in mass of the molecule polymer chains which depend on the vibration frequency under absorption bands. In the P(VDF-HFP) fiber, all absorbance bands for the α-phase were missing while the absorbance peaks at 509 and 840 cm^−1^ were prominent, signifying a strong emergence of the electroactive β-phase. Therefore, the FTIR results demonstrate that electrospinning promotes the transition to β-phase crystals within the P(VDF-HFP) fibers. In addition, the β-phase of the fiber increased with the compression temperature.

An assessment of the relative fraction of the β-phase content, *F*(β), was executed from the IR spectra using the Lambert–Beer law stated in Equation (2). The *F*(β) for all samples is exhibited in Table 1. The P(VDF-HFP) fiber had F(β) ~85.90% exceeding the film by 11.79%. Electrospinning relies on high electric fields and allows the production of sub-micro to nano-scale fibers, with a β-phase fraction up to 86%without any post-treatment. Furthermore, *F*(β) in the fiber increased from 85.90% to 89.65% when compressed at an elevated temperature. This confirms the positive influence of high pressure on β-phase formation as previously reported. Scheinbeim et al. [14] verified that increasing the quenching pressure from 200 to 700 MPa increased the β-phase content in samples from 0% to 85%.

The absolute β fraction (%β) was estimated from the data of both the X_c_ and F(β) with Equation (3) as shown in Table 1. About 36.67 %β was obtained in the film, while the largest 49.33% was obtained with a compression at 80 °C of the fiber mat. The emergence of electroactive β-phase was clearly improved by electrospinning and compression at an elevated temperature which was corroborated by FTIR spectra and XRD patterns.

### 3.4. Thermal Analysis

The study of the thermal behavior was done using the DSC technique. The data are summarized in Table 2. On comparing the P(VDF-HFP) fiber and film, the onset of the melting ($T_{m}^{\mathrm{on}}$) and peak melting ($T_{m}^{p}$) temperatures of the fiber increased with the electrospinning. This indicates that the high electric field (and possibly the dimensions of the sample) influenced crystallization in the sample. For compressed the fiber mats, both $T_{m}^{\mathrm{on}}$ and $T_{m}^{p}$ decreased with compression temperature. In addition, the final melting temperature ($T_{m}^{f}$) in all cases was in the range from 140 to 180 °C, which corresponds to the melting temperatures of the crystalline phases.

In this analysis, the melting enthalpy (ΔH_m_) of compressed P(VDF-HFP) fibers increased with compression temperature, because the particle size increased significantly as seen in the SEM images (Figure 3). Moreover, Madan [34] reported that the specific heat increases as particle size decreases, while the melting entropy and enthalpy decrease. Increased crystallinity can contribute to the mechanical properties of materials. The increased crystallinity may be due to the fact of good interactions and interfacial adhesion between the polymer matrix and the dispersed phase domain surfaces which would also restrict molecular mobility.

The onset crystallization temperature ($T_{c}^{\mathrm{on}}$), peak crystallization temperature ($T_{c}^{p}$), and final crystallization temperature ($T_{c}^{f}$) decreased with the compression temperature. Elevating the compression temperature reduced the P(VDF-HFP) crystallization temperature progressively, indicating a reduced crystallization rate of the P(VDF-HFP) crystals. Besides, the difference, ΔT_c_, increased as the compression temperature decreased. This means that the crystallization rate of the P(VDF-HFP) fibers from the melt was elevated.

### 3.5. Mechanical Properties

**Figure 6:**
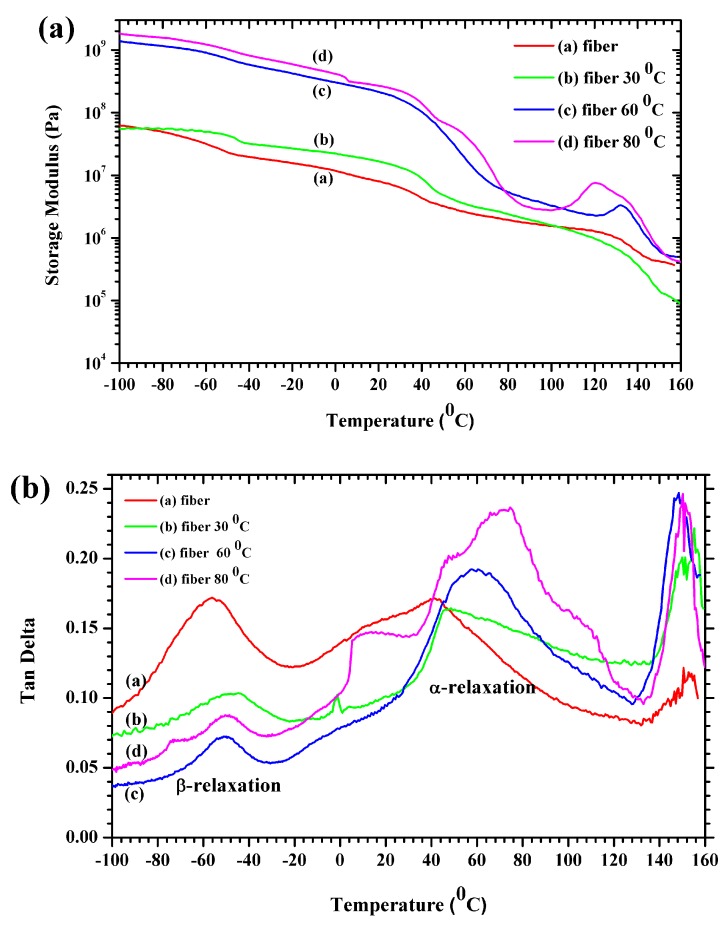
The dynamic mechanical analysis curves of P(VDF-HFP) fiber and compressed fiber mats. (**a**) Storage modulus and (**b**) tan delta.

Dynamic mechanical analysis helps assess the thermomechanical properties and the glass transition temperatures of polymers. The storage modulus (*E*′) and the tan delta as functions of temperature are displayed in Figure 6. The storage modulus decreases with temperature in Figure 6a although not linearly. The storage modulus displays three distinct regions: (1) a glassy high modulus region at low temperatures where the segmental motions are restricted; (2) a transition region with a substantial decrease in *E*′; (3) and a rubbery area with severe decay in the modulus above the glass transition temperature. The storage modulus in both the glassy and rubbery regions increased due to the thermal compression at 30° to 80 °C and was comparatively high in the glassy region relative to the P(VDF-HFP) fiber. The high storage modulus of 80 °C for the compressed P(VDF-HFP) fiber at low temperatures confirms the reinforcement effect at the molecule interfaces. It can be attributed to the restricted molecular mobility in the P(VDF-HFP) fibers by the strengthened interactions with the polymer matrix [35]. A gradual decrease of E′ is observed from −40 to 0 °C which is ascribed to the glass transition of P(VDF-HFP( [36]. It can be seen that the compression temperature influenced the glass transition temperature of the P(VDF-HFP) fibers.

Figure 6b presents the loss tangent (tanδ) as a function of temperature for the P(VDF-HFP) fiber and compressed fiber mats. The dielectric relaxation process can be used to explain the ability motions and cross-linking structure in the amorphous phases and crystalline fraction which are related to the dynamic glass transition. Under the relaxation process at lower than room temperature, the β-relaxation for P(VDF-HFP) fiber was −55 °C which can be obtained from the value of the maximum in the loss tangent. It was found that the board of the β-transition for the P(VDF-HFP) fiber presented from −80 to −20 °C. Moreover, it was clearly shown that the β-transition for the P(VDF-HFP) fibers was lower than the fiber mats. This effect occurred in the cooperative segment’s mobility of the polymer chains in the amorphous regions. Above the room temperature, the damping relaxation process was observed as two peaks. The first peak damping of the relaxation process provided the α-relaxation which was related to the motions in a crystalline fraction [29], while the second peak relaxation process depicted the melting temperature.

Generally, the glass transition temperature (*T*_g_) of a polymeric material is determined from the peak of the tanδ curve [37]. The tanδ has a peak at approximately −40 °C assigned to the glass transition of pure PVDF. In Figure 6b, the glass transition temperatures (*T*_g_), are approximately −56.17, −44.83, −50.83, and −48.97 for the fiber and the 30, 60 and 80 °C compressed fiber mats, respectively. In fact, the *T*_g_ of the polymers was related to the polymer chain′s flexibility. When the rigid regions within the polymer increased, it led to an increase in the value of *T*_g_. In the case of fiber mats, the compression process enhanced the rigidity of the polymer based on the crystallinity fraction or hard segments. However, the *T*_g_ also depended on the heating or cooling rate and the stress rate.

### 3.6. Electrical Properties 

**Figure 7:**
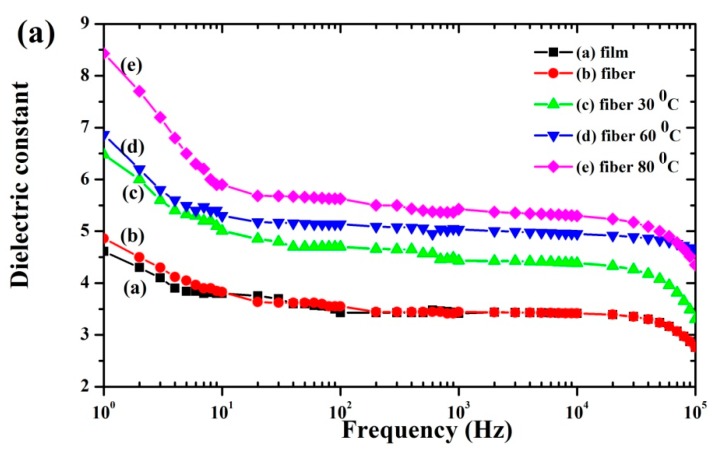

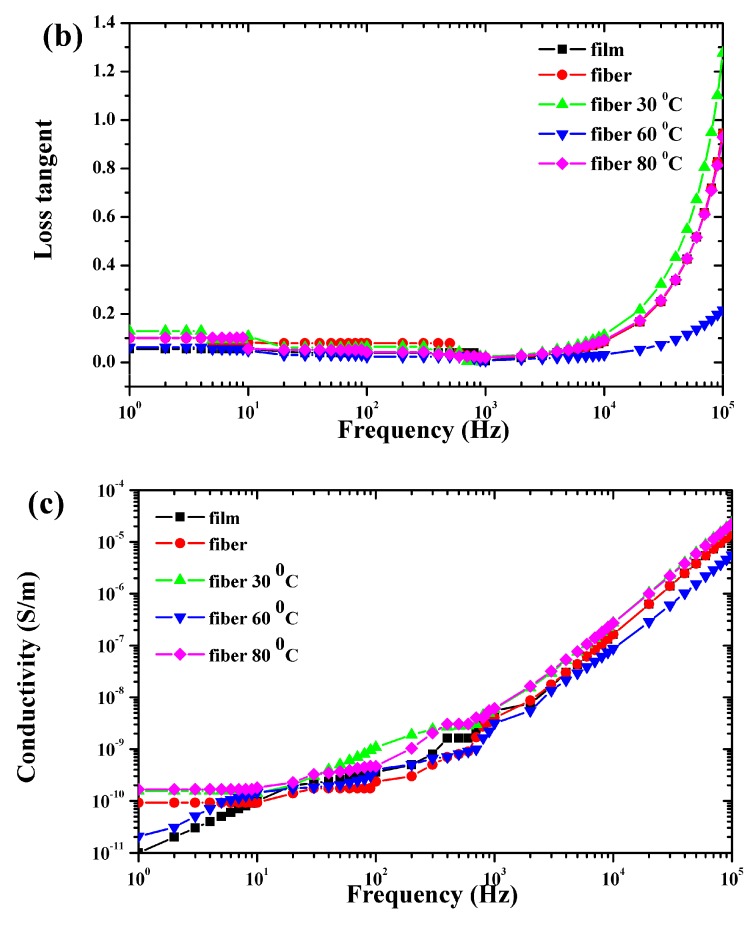
Variation of the dielectric constant (**a**), loss tangent (**b**) and AC conductivity (**c**) with frequencies from 10^0^ to 10^5^ Hz for the film, fibers, and compressed fiber mats.

Figure 7a–c presents the dielectric constant (ε_r_), loss tangent (tanδ), and conductivity (σ)as functions of frequency from 10^0^ to 10^5^ Hz for the P(VDF-HFP) film, fibers, and fiber mats compressed at 30°, 60°, and 80 °C. For all samples (Figure 7a), the dielectric constant decreased with frequency. This was because the dipoles of the dielectric materials cannot follow rapid changes in the field direction [38]. At a high frequency, the dielectric constant then only depends on the dipolar polarization, while the alignment of the dipoles lags behind the field in the polymer matrix. The dielectric constant strongly decreased in the low frequency range up to 20 Hz and then suffered a softer decrease at higher frequencies. This was due to the electric polarization inside the matrix which arises from the electrospinning and compression of the P(VDF-HFP) fibers. 

All samples had the highest dielectric constant at low frequency, which can be explained by the Maxwell–Wagner polarization in a heterogeneous material [39]. Consequently, the organization of filler within the composites or multilayer dielectric, including electrospun fibers, can enhance the Maxwell–Wagner interfacial polarization with surface charge distribution. The maximum dielectric constant was 8.4 at 1 Hz for fiber mats compressed at 80 °C. Clearly, the thermal compression reduced air gaps and added surface charges causing strong Maxwell–Wagner interfacial polarization. 

**Figure 8:**
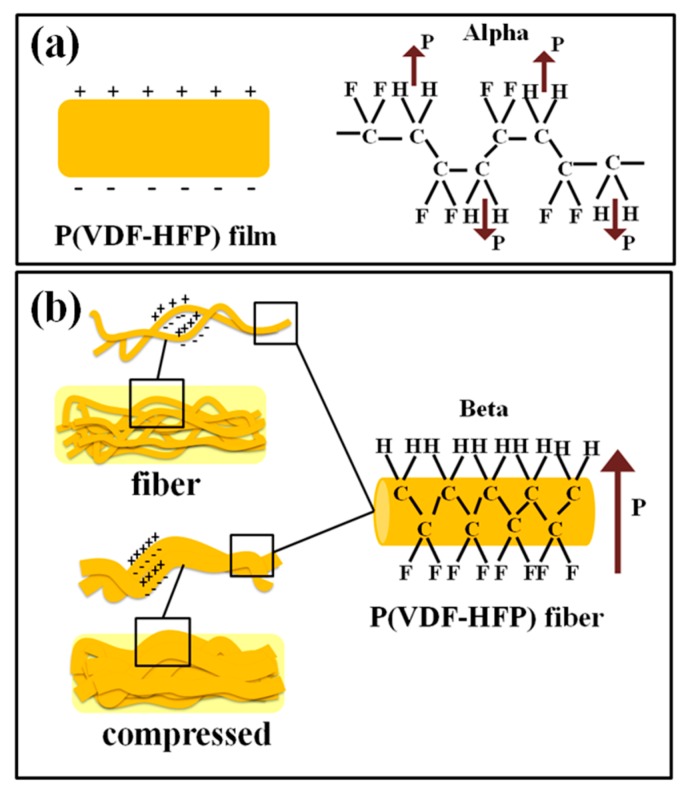
Schematic of the proposed β-phase transformation mechanism.

Interfacial polarization occurs whenever there is an accumulation of charges at interfaces among regions (phases) within a material. Grain boundaries frequently have interfacial polarization, as they can trap charges migrating in an applied field. Dipoles formed by the trapped charges increase the polarization. Interfaces also arise in heterogeneous dielectric materials, for example, when there is a dispersed phase in a continuous matrix. This principle is schematically illustrated in Figure 8. The schematic illustrates the anticipated electroactive β-phase interaction mechanism between the phase and chains of P(VDF-HFP) in the matrix. The P(VDF-HFP) film was prepared without stretching or poling, and the α-P(VDF-HFP) film is shown in Figure 8a. The fibers were then fabricated by electrospinning, thus they contained a β-phase and formed a highly porous fiber mat. Therefore, the interactions among the fiber surfaces were weak because of the air gaps (pores) among the fibers. During thermal compression, the interaction of negatively charged surfaces with C–F and positive –CH_2_ dipoles from (CH_2_–CF_2_) monomers in the P(VDF-HFP) alters the polarity and gives rise to nucleation of the β-phase in fibers [40]. Thus, the compressed fibers had cooperative interactions, apparently with synergistic effects giving an extremely high dielectric constant.

In recent work, the increase in the dielectric constant depended on the crystalline fraction in the polymer [3]. This fraction is normally accompanied by dipole polarization, which increases the melting enthalpy of crystalline domains and can greatly increase the dielectric constant. The observed melting enthalpy and crystallinity were highest for the fiber mats compressed at 80 °C, matching the highest dielectric constant for this case. Moreover, a high specific surface area and overlap without fusing the compressed fibers are the keys to achieving a high dielectric constant [41]. 

Figure 7b presents the dielectric loss versus frequency. Obviously, the dielectric loss increased with the applied frequency. Large dielectric losses are caused by the charges at high frequencies (10^4^ Hz), which is typical owing to the polarization loss and DC conduction loss [42]. On the other hand, the decreased dielectric constant also relates to increased dielectric loss. The AC conductivity in all cases increased at high frequencies, as displayed in Figure 7c. The observed increases in electrical conductivity may be attributed to the polarization of the bound charges [43]. The electrical conductivity linearly increased with frequency, indicating that the number of charge carriers also increased. The electrical conductivity of the fiber mats compressed at 80 °C was the highest, and this might be attributed to the conductive networks formed by the surface contacts in the fibrous matrix and the free electrons within it.

### 3.7. Electrostrictive Properties

**Figure 9:**
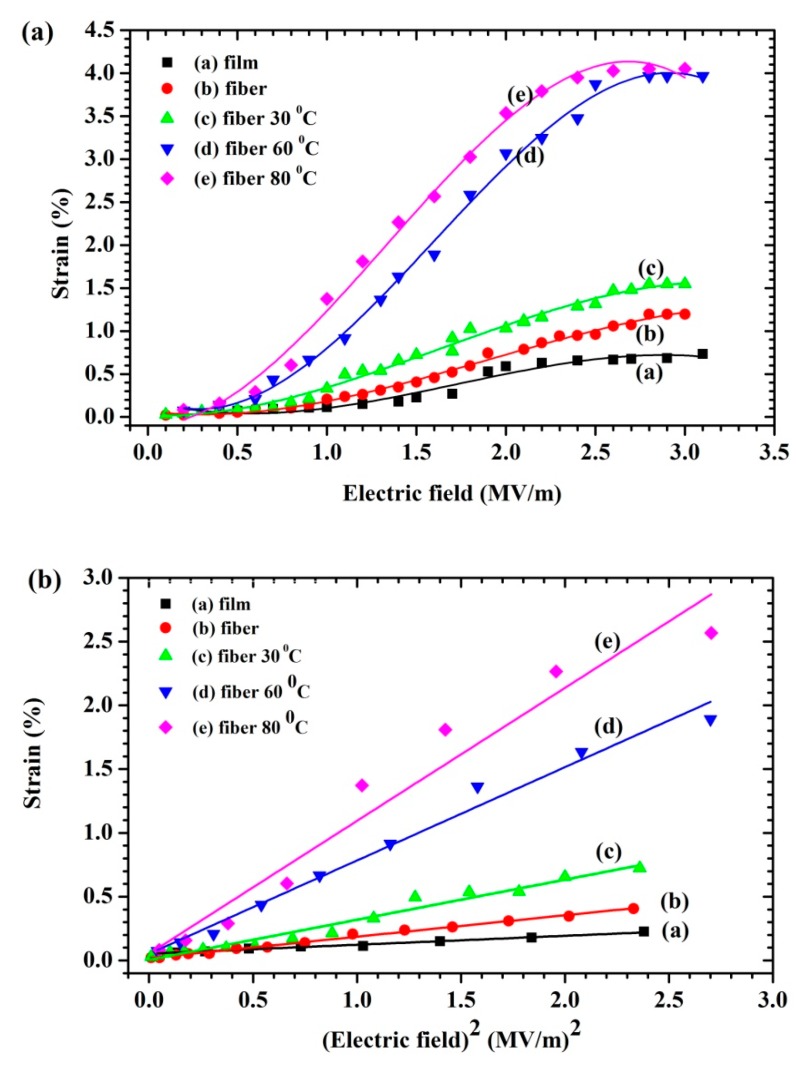

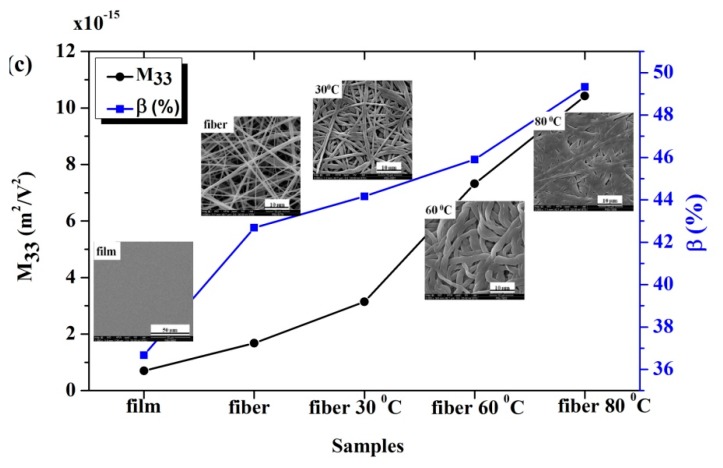
Strain behaviors of film, fibers, and compressed fibers as a function of the (**a**) electric field and (**b**) square of the electric field at 1 Hz. (**c**) The effect of the compression fibers on the electrostrictive coefficients and absolute β fraction.

The longitudinal strain (*S*_3_) behavior of the P(VDF-HFP) film, fibers, and fiber mats compressed at 30°, 60°, and 80°C and induced by an external electric field (*E*_3_) at a frequency of 1 Hz, is presented in Figure 9a. At a low electric field, the electrostriction behavior demonstrated that the total thickness strain had an approximately quadratic relation to the applied electric field. This can be illustrated by the electrostriction which is shown in Equation (7) and Maxwell stress. The Maxwell stress effect involves electrostatic attractions and interactions with the charges on the electrodes showing $S_{M}=\varepsilon_{0}\varepsilon_{r}E_{3}^{2}/Y$. This equation can be used to estimate the value of the Maxwell stress which can be neglected due to its small and negligible value in the case of a low dielectric constant at an applied low electric field. The induced strain of all samples based on the electrostrictive behavior can be observed. The linear relationships between the induced strain and the electric field, shown in Figure 9b, display that their slope can be assigned as the electrostrictive coefficient (*M*_33_). As a result, the electrostrictive behavior of the samples can be expressed according to Equation (7). The $M_{33}$ increased with the fraction of temperatures compressed and the increased number of polymer–polymer interfaces may play a key role, affecting both the electrical and mechanical properties as shown in Figure 9c. This was reflected by the abruptly increased space charge distribution, and the increasing dielectric constant increased with the compressed fibers. Clearly, this study indicates that fibers compressed by temperature are promising electrostrictive materials for actuation and can be fabricated with a simple preparation. 

## 4. Conclusions

In conclusion, electrospinning fibers and thermal compression of the fiber mats enhanced the availability of the interfacial charges, dielectric constant, electroactive β-phase, and electrostrictive coefficient content in P(VDF-HFP). The high electrostatic field in the electrospinning process caused orientation polarization, which apparently helped transform non-polar α-phase to electroactive β-phase in the formed fibers. Increasing the voltage during electrospinning increased the β-phase fraction in fibers from 74.11% to 85.90%. In this study, compressing P(VDF-HFP) fiber mats at 80 °C gave the highest, 89.65%, β-phase fraction among the cases tested. In addition, the dielectric constant and the crystallinity increased with the compression temperature up to 80 °C. This case gave the maximal observed dielectric constant of 8.4 at 1 Hz and also had the largest absolute β fraction (%β) of about 49.33% among the cases tested. Thus, the electrospinning and thermal compressing coupling processes can enhance the induced interfacial polarization, and β-phase leads to the high electrostrictive properties of obtained P(VDF-HFP) nanofibers.

## Figures and Tables

**Table 1:** Analysis of the β-phase fraction in the crystalline region of the samples.

Sample	*X_c_*(%)	*A*_β_ (cm^−1^)	*A*_α_ (cm^−1^)	*F*(β) (%)	%β
Film	49.47	0.3161	0.0875	74.11	36.67
Fiber	49.69	0.1353	0.0176	85.90	42.68
Fiber 30 °C	50.88	0.1569	0.0189	86.80	44.16
Fiber 60 °C	52.44	0.2632	0.0297	87.53	45.90
Fiber 80 °C	55.03	0.327	0.0299	89.65	49.33

**Table 2:** Thermal properties of the samples.

Sample	$T_{m}^{\mathrm{on}}$	$T_{m}^{p}$	$T_{m}^{f}$	Δ*T*_m_	Δ*H*_m_	$T_{c}^{\mathrm{on}}$	$T_{c}^{p}$	$T_{c}^{f}$	Δ*T*_c_	Δ*H*_c_
Film	132.4	158.3	170.5	38.1	21.0	140.5	136.4	131.2	9.3	−26.6
Fiber	136.0	160.1	170.1	34.1	22.7	140.9	137.1	131.7	9.2	−25.2
Fiber 30 °C	143.0	159.0	170.8	27.8	25.2	142.2	136.4	130.8	11.4	−27.0
Fiber 60 °C	137.2	158.3	171.8	34.6	29.3	139.1	134.3	129.1	10.0	−27.0
Fiber 80 °C	135.5	158.2	170.9	35.4	36.3	138.6	134.2	129.6	9.0	−26.2

$T_{m}^{\mathrm{on}}$: onset melting temperature; $T_{m}^{p}$: peak melting temperature; $T_{m}^{f}$: final melting temperature; ${\Delta T}_{m}=T_{m}^{f}-T_{m}^{\mathrm{on}}$; ${\Delta H}_{m}$: melting enthalpy; $T_{c}^{\mathrm{on}}$: onset crystallization temperature;.$T_{c}^{p}$: peak crystallization temperature; $T_{c}^{f}$: final crystallization temperature; ${\Delta T}_{c}=T_{c}^{\mathrm{on}}-T_{c}^{f}$; ${\Delta H}_{c}$: crystallization enthalpy.
